# Modifiable risk factors for ventilator associated diaphragmatic dysfunction: a multicenter observational study

**DOI:** 10.1186/s12890-023-02633-y

**Published:** 2023-09-13

**Authors:** Hong Pu, Gordon S. Doig, Yu Lv, Xiaoxiao Wu, Fu Yang, Shurong Zhang, Zongan Liang, Yan Zhou, Yan Kang

**Affiliations:** 1 Department of Critical Care Medicine, West China Medical School, West China Hospital, Sichuan University, Chengdu, PR China; 2 Northern Clinical School Intensive Care Research Unit, Sydney Medical School, University of Sydney, Sydney, NSW Australia; 3 Healthcare-Associated Infection Control Center, Sichuan Academy of Medical Sciences, School of Medicine, Sichuan People’s Hospital, University of Electronic Science and Technology of China, Chengdu, PR China; 4 Department of Critical Care Medicine, Sichuan Academy of Medical Sciences, School of Medicine, Sichuan People’s Hospital, University of Electronic Science and Technology of China, Chengdu, PR China; 5 Department of Respiratory and Critical Care Medicine, West China Medical School, West China Hospital, Sichuan University, Chengdu, PR China

**Keywords:** Respiration, Artificial, Ventilator weaning, Risk factors, Amino acids, Causality

## Abstract

**Background:**

Diaphragmatic dysfunction is known to be associated with difficulties weaning from invasive mechanical ventilation and is related to worse patient outcomes yet our understanding of how to prevent diaphragmatic dysfunction remains incomplete. We examined potentially modifiable risk factors for diaphragmatic dysfunction and attempted to estimate benefits attributable to altering these modifiable risk factors.

**Methods:**

This prospective multicenter observational study was undertaken in the general ICUs of two tertiary care teaching hospitals. Critically ill adults expected to receive invasive mechanical ventilation for at least 48 h were enrolled. Diaphragm function was assessed by ultrasound each study day, with dysfunction defined as thickening fraction less than 20%.

**Results:**

From January to December 2019, 856 patients were screened and 126 patients were enrolled. Overall, 40.5% (51/126) of patients experienced diaphragmatic dysfunction during invasive mechanical ventilation. Patients with diaphragmatic dysfunction were more likely to develop ventilator associated pneumonia (risk difference [RD] + 12.9%, 95% Confidence Interval [CI] 1.4 to 24.4%, *P* = 0.028), were more likely to experience extubation failure (RD + 8.5%, 95% CI 0.4 to 16.6%, *P* = 0.039) and required a longer duration of invasive mechanical ventilation (RD + 1.3 days, 95% CI 0.1 to 2.5 days, *P* = 0.035). They also required a longer hospital stay (RD + 1.2 days, 95% CI 0.04 to 2.4 days, *P* = 0.041) and were more likely to die before hospital discharge (RD + 18.1%, 95% CI 3.7 to 32.5%, *P* = 0.014). Multivariable analysis considered the impact of age, sex, pre-existing nutritional status, caloric intake, amino acid intake, acute disease severity, modes of mechanical ventilation, measures of respiratory status, sedation, pain control and baseline diaphragm thickness. Only SOFA score (*P* = 0.008) and early amino acid intake (*P* = 0.001) remained significant independent risk factors for the onset of diaphragmatic dysfunction. Causal path modeling suggested early amino acid intake may significantly reduce diaphragmatic dysfunction (RRR 29%, 95% CI 10% to 48%, *P* = 0.003) and may also reduce mortality (RRR 49%, 95% CI 25% to 73%, *P* < 0.0001).

**Conclusions:**

Amino acid intake during the first 24 h of ICU stay may represent an important, modifiable risk factor for diaphragmatic dysfunction and may have a direct causal effect on mortality. We recommend additional research on this topic.

## Introduction

Worldwide, 13 to 20 million critically ill patients require mechanical ventilation to sustain their respiratory function every year [1]. Up to 46% of these patients have difficulties being weaned from mechanical ventilation once respiratory support is no longer needed [2]. Furthermore, patients who have ventilation prolonged due to difficulties weaning are more likely to develop pulmonary complications, require a longer duration of hospital stay and are more likely to die before hospital discharge [3–5].

Failure to successfully wean from a ventilator may arise from a general loss of muscle strength due to extended critical illness with conditions like sepsis [6]. Use of systemic corticosteroids [7] and neuromuscular blocking agents [8] have also been associated with difficulties weaning from mechanical ventilation. The impact of each of these factors can be further potentiated by general malnutrition arising from inadequate nutritional intake [9]. One pathway common to all of the above processes is loss of diaphragm function, which directly impacts the patient’s ability to sustain normal unassisted breathing [10, 11].

To understand the impact of diaphragm function on prolonged ventilator dependence, Goligher et al. conducted a prospective multicenter observational study enrolling 211 critically ill patients who required mechanical ventilation [3]. On each day of intensive care unit (ICU) stay, diaphragm function was assessed using ultrasound imaging and it was found that a loss of diaphragm function was significantly associated with difficulties weaning from mechanical ventilation, prolonged ICU admission and increased the risk of complications. Although it was hypothesized that critically ill patients may benefit from interventions targeted towards maintaining diaphragm function during mechanical ventilation, because of the nature of their study design, they were not able to estimate the magnitude of potential benefits arising from modifying risk factors for loss of diaphragm function.

We conducted this prospective multicenter observational study to examine modifiable risk factors for the loss of diaphragm function in mechanically ventilated critically ill patients and to estimate potential benefits arising from modification of these risk factors.

## Materials and methods

### Study cohort and setting

This study was conducted in the general ICUs of the West China Hospital of Sichuan University and the Sichuan Provincial People’s Hospital, University of Electronic Science and Technology of China. Institutional Review Board approval was obtained (West China Hospital of Sichuan University Human Ethics Committee Protocol No. 133/2019) and written informed consent was provided by participants or their surrogates. The study followed the Strengthening the Reporting of Observational Studies in Epidemiology (STROBE) statement [12] and the protocol was registered with the Chinese Clinical Trials Registry as an observational study (ChiCTR1900022188).

From January to December 2019, adult patients were screened within 24 h of intubation to ascertain whether they were expected to remain mechanically ventilated for at least 48 h. Exclusion criteria included: 1) history of diaphragmatic disease or surgery; 2) multiple rib fractures; 3) invasive mechanical ventilation in the last 6 months; 4) neuromuscular disease; 5) use of neuromuscular blockers; 6) chronic obstructive pulmonary disease; 7) intubation for sepsis or septic shock; 8) inability to perform dialysis; 9) expected brain death; 10) planned palliative care only or; 11) inability to obtain informed consent.

Demographic data, illness severity scores, comorbidities, amino acid intake, calorie intake, ventilator modes, oxygenation, sedation scores, pain scores and patient outcomes were prospectively recorded.

### Ultrasound technique

Ultrasonography was performed daily using a CX50 portable ultrasound (Philips, USA) equipped with a high resolution 10-MHz linear probe and a 7·5-MHz convex phased-array probe. Right diaphragm thickness was measured in the zone of apposition at the level of the ninth or tenth intercostal space near the midaxillary line using a previously validated technique [13, 14].

Diaphragm thickness was measured every study day by two trained operators. Each operator obtained complete measurements daily, independent of the other operator, and these results were averaged for daily analysis. If Spontaneous Breathing Tests (SBT) were performed, ultrasound measurement was always conducted during the SBT when breathing was stable at about 5 min after SBT initiation. If SBT was not performed, ultrasound measurement was conducted at any time respiratory status was stable (i.e. oxygenation, respiratory rate and rhythm were constant, there was no patient-ventilator asynchrony and the patient was not actively coughing).

Ventilator parameters were set by a respiratory therapist during SBT as follows: fraction of inspired oxygen (FiO_2_) 40% to 50%, positive end-expiratory pressure (PEEP) 5 to 8 cmH_2_O and Pressure Support 8 to 12 cmH_2_O [15]. Individual clinician preferences were used to set specific parameters for specific patients. Eligibility for SBT was judged daily by the attending physician based on the following criteria: PaO2 to FiO_2_ ratio ≥ 150 mmHg**;** PEEP ≤ 5 to 8 cmH2O; pH ≥ 7·25; stable hemodynamics and; adequate cough [16].

### Definition of diaphragmatic dysfunction

Diaphragmatic dysfunction was defined as diaphragmatic thickening fraction (TF) of less than 20% [17] at any time during the study. TF was calculated as follows [17]:

$$\mathrm{TF}=(\mathrm{End}\; \mathrm{inspiratory}\;\mathrm{thickness}-\mathrm{End}\;\mathrm{expiratory}\;\mathrm{thickness})/\mathrm{End}\;\mathrm{expiratory}\;\mathrm{thickness}\times100$$

Only the first incident of diaphragmatic dysfunction was used in analysis.

### Definition of extubation failure

Extubation failure was defined as reintubation *and* recommencement of invasive mechanical ventilation within 48 h of attempted liberation from invasive mechanical ventilation by extubation [18]. Study measurements were restarted if extubation failure occurred.

### Statistical analyses

Analyses were conducted using SAS Version 9.4 (SAS Institute). Univariate logistic regression was used to investigate the relationship between exposure variables and subsequent diaphragmatic dysfunction. Variables with univariate *P*-value < 0.10 were eligible for assessment in a maximum covariate adjusted logistic regression model. Stability of the maximum model was assessed using Eigenanalysis with a Condition Number greater than 30 considered to indicate the presence of moderate to severe multicollinearity. If moderate to severe multicollinearity was present, standardization (z-transformation) of continuous variables was undertaken before backwards elimination commenced. Variables were removed from the maximum covariate adjusted model one-by-one if their multivariable *P*-value was greater than 0.10. Next, using all variables remaining in the covariate adjusted model, patient-centered benefits attributable to modifiable risk factors were assessed using path analysis (SAS Proc CALIS). A two-sided *P*-value less than 0.05 was accepted to indicate statistical significance.

## Results

**Fig. 1:**
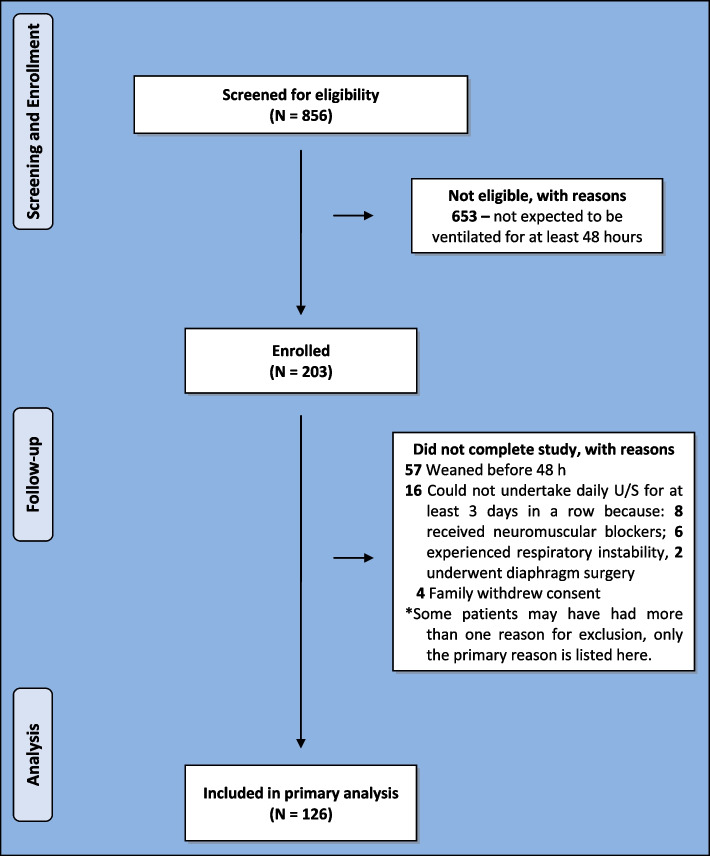
Flow diagram of the patient selection process. Legend: U/S: ultrasound

During the 12-month study period, 856 patients were screened for eligibility within 24 h of intubation. Of these 856 patients, 203 patients were expected to be ventilated for longer than 48 h so they were consented and received an initial ultrasound. Fifty-seven of these 203 patients were weaned within the next 24 h and 16 patients could not could not complete daily ultrasound assessments for more than three days in a row (8 patients received neuromuscular blockers during mechanical ventilation, 6 patients experienced severe respiratory instability, and 2 patients underwent diaphragm surgery during the study period). In addition, 4 patients` families withdrew consent for participation. Therefore 126 patients completed the study and were able to be analyzed. See Fig. 1 for additional information on patient flow.

The mean (SD) age of study patients was 58.7 (17.2) years and 61.9% (78/126) were male. Over the first 24 h of ICU admission, the mean APACHE II score was 18.7 (6.1) and the mean SOFA score was 8.9 (4.6). Seventy percent (88/126) of patients were admitted to ICU for medical reasons whilst the remaining thirty percent (38/126) of patients were admitted after surgery. The overall hospital discharge mortality rate was 20.6% (26/126) and 96% (121/126) of patients underwent SBT at some time during their ICU stay. Forty percent (51/126) of patients developed loss of diaphragmatic dysfunction at some time during mechanical ventilation. See Table 1 for complete baseline information.

**Table 1:** Risk factors for the onset of diaphragmatic dysfunction

Variable	All patients (*n* = 126)	Diaphragmatic dysfunction (*n* = 51)	No diaphragmatic dysfunction (*n* = 75)	OR for developing diaphragmatic dysfunction	*P*-value*
Age years, mean (SD)	58.6 (17.2)	60.8 (15.1)	57.2 (18.5)	1.012	0.255
Male percent (n/N)	61.9% (78/126)	56.8% (29/51)	65.3% (49/75)	0.699	0.337
BMI kg/m^2^, mean (SD)	22.1 (2.7)	21.9 (3.0)	22.3 (2.5)	0.945	0.402
Medical vs. Surgical admission percent, n/N	69.8% (88/126)	72.5% (37/51)	68.0% (51/75)	1.244	0.585
APACHE II^a^ mean (SD)	18.7 (6.1)	20.0 (5.8)	17.8 (6.3)	1.059	0.060
SOFA^a^ mean (SD)	8.9 (4.6)	10.4 (6.8)	7.9 (4.1)	1.138	0.003
AA intake^a^ g/kg/day, mean (SD)	0.92 (0.25)	0.82 (0.28)	0.98 (0.21)	0.054	0.0006
Caloric intake^a^ kcal/kg/day, mean (SD)	11.8 (7.8)	10.3 (6.8)	12.8 (8.4)	0.958	0.086
Volume Control Ventilation vs. Other Modes^b^ percent (n/N)	96.0% (121/126)	92.2% (47/51)	98.7% (74/75)	0.159	0.105
Highest PEEP^a^ cmH_2_O, mean (SD)	6.9 (2.1)	7.1 (2.4)	6.8 (1.9)	1.077	0.393
Lowest PaO_2_:FiO_2_ ratio^a^ mean (SD)	216 (113)	202 (99)	225 (121)	0.998	0.284
Baseline diaphragm thickness mm, mean (SD)	2.40 (0.66)	2.38 (0.60)	2.42 (0.70)	0.907	0.729
Sedation, RASS ≤ -3 (n/N)	81.7% (103/126)	84.3% (43/51)	80% (60/75)	1.344	0.704
Pain control, CPOT < 3, (n/N)	91.3% (115/126)	94.1% (48/51)	89.3% (67/75)	1.910	0.540

*OR* Odds ratio, *BMI* Body mass ratio, *APACHE* Acute physiology and chronic health evaluation, *SOFA* Sequential organ failure assessment, *AA* Amino acids, *PEEP* Positive end-expiratory pressure, *RASS* Richmond Agitation Sedation Scale (≤ -3 indicates at least moderate sedation); CPOT: Critical Care Pain Observation Tool (CPOT < 3 indicates acceptable levels of pain)

^a^Collected over first 24 h in intensive care unit

^b^Volume Control Ventilation vs. all Other Modes (Ex. Pressure Control, Synchronized Intermittent Mandatory Ventilation, Spontaneous breaths etc.) over first 24 h in intensive care unit

^*^P-value from univariate logistic regression

Patients who developed diaphragmatic dysfunction were significantly more likely to experience new onset sepsis (absolute risk difference + 12.2%, 95% CI 1.4 to 23.0%, *P* = 0.026) or ventilator associated pneumonia (absolute risk difference + 12.9%, 95% CI 1.4 to 24.4%, *P* = 0.028) and were significantly more likely to die before hospital discharge (absolute risk difference + 18.1%, 95% CI 3.7 to 32.5%, *P* = 0.014). Furthermore, they required more time on the ventilator (mean difference + 1.3 days, 95% CI 0.1 to 2.5 days, *P* = 0.035), were more likely to receive a tracheotomy (absolute risk difference + 11.0%, 95% CI 1.0 to 21.0%, *P* = 0.030) and were more likely to experience extubation failure (absolute risk difference + 8.5%, 95% CI 0.4 to 16.6%, *P* = 0.039). Overall, they also had a significantly longer ICU stay (mean difference + 1.8 days, 95% CI 0.1 to 3.5 days, *P* = 0.037) and a significantly longer hospital stay (mean difference + 1.2 days, 95% CI 0.04 to 2.4 days, *P* = 0.041). See Table 2 for additional details of outcomes associated with diaphragmatic dysfunction.

**Table 2:** Associations between clinical outcomes and diaphragmatic dysfunction

Outcome	All patients (*n* = 126)	Diaphragmatic dysfunction (*n* = 51)	No diaphragmatic dysfunction (*n* = 75)	*P*-value*
Death before hospital discharge percent, n/N	21.6% (26/126)	31.4% (16/51)	13.3% (10/75)	0.014
Sepsis percent, n/N	10.3% (13/126)	17.6% (9/51)	5.3% (4/75)	0.026
Extubation failure percent, n/N	4.8% (6/126)	9.8% (5/51)	1.3% (1/75)	0.039
Tracheotomy percent, n/N	7.1% (9/126)	13.7% (7/51)	2.7% (2/75)	0.030
Ventilator associated pneumonia percent, n/N	11.9% (15/126)	19.6% (10/51)	6.7% (5/75)	0.028
Continuous renal replacement therapy percent, n/N	3.2% (4/126)	5.9% (3/51)	1.3% (1/75)	0.154
Duration of ventilation days, mean (SD)	5.9 (5.1)	5.9 (3.7)	4.6 (3.0)	0.035
Duration of ICU stay days, mean (SD)	8.1 (7.19)	8.1 (5.9)	6.3 (3.5)	0.037
Duration of hospital stay days, mean (SD)	14.9 (10.0)	22.3 (12.9)	21.1 (9.4)	0.041

*SD* Standard deviation, *ICU* Intensive care unit

^*^From univariate analysis

At study baseline, there was a difference between patients who developed diaphragmatic dysfunction and those who did not with regards to APACHE II score (mean difference + 1.2, 95% CI -0.05 to 2.5, *P* = 0.060) and SOFA score (mean difference + 2.5, 95% CI 0.9 to 4.1, *P* = 0.003). Furthermore, patients who developed diaphragmatic dysfunction received less amino acids (mean difference -0.16 g/kg/day, 95% CI -0.25 to 0.07 g/kg/day, *P* = 0.0006) but did not differ significantly with regards to energy intake (mean difference -2.5 kcal/kg/day, 95% CI -5.4 to 0.35 kcal/kg/day, *P* = 0.086) over the first 24 h of ICU admission. There was no significant difference in the baseline diaphragm thickness of patients who developed diaphragmatic dysfunction compared to those who did not (mean difference -0.04 mm, 95% CI -0.65 to 0.57 mm, *p* = 0.907). No other baseline characteristics differed between the two groups. See Table 1 for complete details of differences between groups.

Multivariable logistic regression was conducted to control for baseline differences in severity of illness (APACHE II score and SOFA score), caloric intake and amino acid intake over the first 24 h of ICU admission. Initial stability assessment of the maximum covariate adjusted model revealed the presence of severe multicollinearity (Condition Number = 164). After standardization (z-transformation) of all continuous variables (APACHE II, SOFA, caloric intake and amino acid intake) the Condition Number was reduced to 3.4, indicating that instability due to multicollinearity had been addressed.

Stepwise backwards elimination resulted in the removal of caloric intake (OR 0.874, 95% CI 0.579 to 1.321, *P* = 0.523) and APACHE II score (OR 1.264, 95% CI 0.804 to 1.987, *P* = 0.310) from the covariate adjusted model. Only SOFA score (OR 1.787, 95% CI 1.163 to 2.745, *P* = 0.008) and amino acid intake (OR 0.490, 95% CI 0.318 to 0.756, *P* = 0.001) during the first 24 h of ICU admission remained significant independent predictors of diaphragmatic dysfunction. See Table 3 for complete details of the multivariable analysis.

**Table 3:** Multivariable logistic regression analysis of risk factors for diaphragmatic dysfunction

Variable	Regression parameter estimate	OR (95% CI) for developing diaphragmatic dysfunction	*P*-value*
Caloric intake^a^ kcal/kg/day	-0.1344	0.874 (0.579 to 1.321)	0.523^**‡**^
APACHE II^a^	0.2344	1.264 (0.804 to 1.987)	0.320^**‡**^
SOFA^a^	0.5803	1.787 (1.163 to 2.745)	0.008^**§**^
AA intake^a^ g/kg/day	-0.7131	0.490 (0.318 to 0.756)	0.001^**§**^

*OR* Odds ratio, *95% CI* 95% Confidence interval, *APACHE* Acute physiology and chronic health evaluation, *SOFA* Sequential organ failure assessment, *AA* Amino acids

^a^Collected over first 24 h in ICU

^*^From multivariable logistic regression

^**‡**^Variable eliminated from final model due to multivariable *P*-value > 0.10

^**§**^Variable retained in final model

**Fig. 2:**
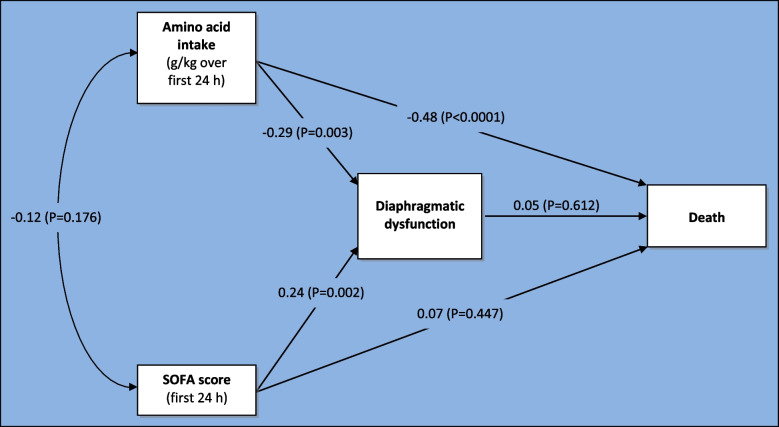
Causal pathway analysis assessing impact of amino acid intake and SOFA score on mortality. Legend: *Standardized path coefficients obtained by maximum likelihood estimation using SAS Ver 6.4, Proc CALIS; SOFA: Sequential Organ Failure Assessment score

To assess potential patient benefits, causal path analysis was conducted using all significant independent predictors of outcome. Controlling for SOFA score, amino acid intake during the first 24 h of ICU stay was found to significantly reduce the onset of diaphragmatic dysfunction (29% relative risk reduction [RRR], 95% Confidence Interval [CI] 10% to 48%, *P* = 0.003) and mortality (49% RRR, 95% CI 25% to 73%, *P* < 0.0001). No causal relationships with ICU or hospital stay were revealed. See Fig. 2 and Table 4 for additional information on path analysis.

**Table 4:** Path analysis: standardized direct and indirect effects on outcomes

**Outcome**	**Independent variable**	**Total effect** 95% CI *(P-value)*	**Direct effect** 95% CI *(P-value)*	**Indirect effect** 95% CI *(P-value)*
Death before hospital discharge	Amino acid intake	**-0.4898** -0.7346 to -0.2450 *(P* < *0.0001)*	**-0.4759** -0.7138 to -0.2381 *(P* < *0.0001)*	**-0.0140** -0.0285 to 0.0493 *(P* = *0.613)*
	SOFA score	**0.0825** -0.0868 to 0.2518 *(P* = *0.345)*	**0.0710** -0.1086 to 0.2506 *(P* = *0.447)*	**0.0115** -0.0321 to 0.0551 *(P* = *0.618)*
Duration of ICU stay	Amino acid intake	**0.0818** -0.0781 to 0.2418 *(P* = *0.321)*	**0.0916** -0.0690 to 0.2522 *(P* = *0.267)*	**-0.0098** -0.0650 to 0.0454 *(P* = *0.741)*
	SOFA score	**0.0108** -0.1050 to 0.1266 *(P* = *0.865)*	**0.0027** -0.1250 to 0.1304 *(P* = *0.970)*	**0.0081** -0.0366 to 0.0528 *(P* = *0.736)*
Duration of hospital stay	Amino acid intake	**0.0854** -0.0635 to 0.2343 *(P* = *0.264)*	**0.0577** -0.0822 to 0.1976 *(P* = *0.427)*	**0.0297** -0.0288 to 0.0883 *(P* = *0.325)*
	SOFA score	**0.0015** -0.1310 to 0.1340 *(P* = *0.984)*	**0.0259** -0.1348 to 0.1867 *(P* = *0.765)*	**-0.0244** -0.0762 to 0.0274 *(P* = *0.362)*

*ICU* Intensive care unit, *SOFA* Sequential organ failure assessment

### Sensitivity analysis

Sensitivity analysis conducted using only the 121 patients who underwent SBT did not change our primary conclusions.

SOFA score (*P* = 0.009) and amino acid intake (*P* = 0.002) during the first 24 h of ICU admission remained the only significant independent predictors of diaphragmatic dysfunction. Causal path analysis conducted using all significant independent predictors of outcome found that, controlling for SOFA score, amino acid intake during the first 24 h of ICU stay significantly reduced the onset of diaphragmatic dysfunction (28% RRR, 95% CI 8% to 48%, *P* = 0.005) and significantly reduced mortality (44% RRR, 95% CI 22% to 66%, *P* < 0.0001). No causal relationships with ICU or hospital stay were revealed.

## Discussion

In this multicenter observational study, we sought to identify modifiable risk factors for loss of diaphragmatic function during mechanical ventilation. To achieve this, we studied 126 critically ill patients who were ventilated for at least 48 h and used ultrasound imaging to prospectively identify patients who experienced diaphragmatic dysfunction. We found that patients who experienced diaphragmatic dysfunction were eight times more likely to develop extubation failure, required longer ICU stays and were two times more likely to die. Furthermore, we identified amino acid intake during the first 24 h of ICU stay as an independent predictor of the onset of diaphragmatic dysfunction. Causal path analysis revealed that amino acid intake during the first 24 h of ICU stay could reduce the relative risk of developing diaphragmatic dysfunction by 10 to 48% and could reduce the relative risk of mortality by 25 to 73%.

Major cohort studies report that 25 to 45% of critically ill patients fail their first attempt at weaning from mechanical ventilation [2, 18]. These patients require a significantly longer ICU stay and have an increased risk of death [18]. Although the onset of diaphragm weakness is emerging as a key factor influencing weaning success [19, 20], because diaphragm function is not routinely measured during ICU stay, systematic reviews of observational studies assessing risk factors for weaning success often do not emphasise the importance of diaphragm function [21]. In our study, we found that risk of extubation failure increased by eight times after the onset of diaphragmatic dysfunction. Diaphragmatic dysfunction was also significantly associated with increased mortality, duration of ventilation, ICU stay and hospital stay (Table 2). In the context of previous studies that have demonstrated an association between diaphragmatic dysfunction and mortality [5], our findings highlight the need for a greater focus on the role of diaphragm function and weaning success.

After considering the influence of patient characteristics such as age, sex, pre-existing nutritional status, caloric intake, acute disease severity, modes of mechanical ventilation, measures of respiratory status, sedation, pain control and baseline diaphragm thickness (Table 1), we found that SOFA score and amino acid intake during the first 24 h of ICU stay were the only significant independent risk factors for the onset of diaphragmatic dysfunction (Table 3). In adults with cystic fibrosis, worsening nutritional status is strongly related to decreased diaphragm strength [22]. In critical illness, muscle wasting and diaphragmatic dysfunction are known to be associated with each other [5] and increased catabolic activity (autophagy) can be seen in the diaphragm after only 15 h of mechanical ventilation [23]. It is widely accepted that starvation induces the onset of autophagy and that autophagy is down-regulated within 20 min of exposure to amino acids [24]. A large-scale randomised controlled trial conducted in critically ill patients demonstrated that early nutrition support, commenced within 24 h of ICU admission, protected against muscle wasting and reduced duration of mechanical ventilation [25]. A more recent clinical trial that evaluated later increases in protein intake, commenced up to 96 h after ICU admission, failed to show any clinical benefits [26]. Given the known early onset of autophagy in the diaphragm [24], it is entirely plausible that early amino acid intake is required to help mitigate the onset of subsequent diaphragmatic dysfunction [27].

Path analysis is a form of multiple regression analysis that seeks to understand relationships between a dependent outcome variable and two or more related independent variables to test the strength of a hypothesised set of causal relationships [28]. Path analysis is also known as causal modelling or analysis of covariance structures. In our path analysis model, we hypothesised a two tier model whereby covariates (SOFA score and early amino acid intake) could influence each other and could also influence outcome directly and indirectly through modifying diaphragmatic dysfunction (Fig. 2). We included only SOFA score and early amino acid intake in our path analysis because they were the only two independent predictors of diaphragmatic dysfunction detected by multivariable logistic regression. Our path analysis model found both SOFA score and early amino acid intake may have significant causal relationships with the onset of diaphragmatic dysfunction but only early amino acid intake appeared to have a direct impact on mortality. The magnitude of this impact on mortality [RRR 49%, 95% CI 25 to 73% RRR] is similar to the magnitude [37% RRR, 95% CI 3 to 71%] reported in a subgroup analysis of a randomised controlled trial comparing early amino acid intake in critical illness to standard care [27]. Failure to find a direct causal impact of SOFA on mortality is novel and may have arisen due to the relatively small size of our dataset and lack of power. Furthermore, our path analysis models did not find evidence of a similar causal impact on ICU or hospital length of stay. These direct and indirect causal relationships need to be confirmed by future well-conducted and appropriately powered studies.

The loss of diaphragm function occurs gradually over time during mechanical ventilation [29] and, in epidemiological terms, is considered to be a *time-varying covariate*. With a time-varying covariate and a time-related outcome like ICU and hospital stay, inferring causality becomes complex. To detect unbiased associations between time-varying covariates and time-related outcomes, future studies may need to be quite large or match patients who experience diaphragmatic dysfunction to control patients with similar chronic comorbidities, acute disease severity and other relevant characteristics at time of onset [21]. A lack of matching to account for the time-varying nature of diaphragmatic dysfunction and time-related outcomes such as ICU or hospital stay may explain why our study failed to demonstrate a relationship with these outcomes and why other studies have failed to establish associations between diaphragmatic dysfunction and ICU or hospital stay [5]. Relationships between time-varying risk factors and outcomes are complex. Large observational studies, or well conducted randomised controlled trials that *prevent* the onset of diaphragmatic dysfunction, may be needed to understand these complexities.

### Strengths and limitations

Our study was prospective, however, it was conducted at just two sites and enrolled only 126 patients. Although we employed objective statistical screening methods appropriate for detecting potential confounders in small datasets, failure to detect relationships between many variables (Ex. Oxygenation, ventilation modes, BMI etc.) and diaphragmatic dysfunction may have occurred due to lack of statistical power. Because of this issue, our final covariate adjusted model which contained only two variables (SOFA and amino acid intake), must be interpreted conservatively. However, our results do highlight the need for further study across multiple sites with diverse patient populations.

Key definitions used in our study were standardized across sites and were selected because of their acceptance in the published literature. For example, although there are ongoing discussions about how to best classify the outcomes of liberation from invasive mechanical ventilation, need for reintubation and recommencement of ventilator support within 48 h of extubation remains the most commonly used definition of failure [18]. Furthermore, the definition of diaphragmatic dysfunction employed in our study (loss of thickening fraction) was used in the earliest study in this field [10] and has been employed by recent landmark studies of critically ill patients [3, 17]. One important issue arising during critical illness is the inability to assess diaphragmatic thickening during a period of maximum inspiratory effort unless SBT for weaning is being performed. In our study, only five of 126 participants did not undergo an SBT and sensitivity analysis excluding these five patients demonstrated this had no impact on our results. Furthermore, it is recognized that over-assistance during pressure support ventilation may result in a loss of diaphragmatic thickening fraction. Ninety-six percent (121/126) of patients in our study received volume control ventilation during their ICU stay and ventilation mode was not found to influence loss of diaphragmatic thickening fraction (Table 1, *P* = 0.11).

One strength of our observational study is that it attempted to go a step beyond just detecting associations. By conducting path analysis to assess the potential causal impact of a modifiable risk factor, estimates of benefit were obtained which can be used to scope sample sizes required for subsequent interventional studies.

Path analysis revealed that early amino acid intake may have a strong causal impact on mortality, however, our study assessed only one pathway through which this may occur (prevention of diaphragmatic dysfunction). Although other plausible mechanisms through which early amino acid intake may influence mortality have been proposed [30], they were not the focus of this current study.

A final important limitation when considering our study findings is that it was conducted pre-COVID-19. Given the virus may have a direct effect on diaphragmatic function [31], it is possible assessment of diaphragmatic function in critically ill patients is more important post-COVID-19 [32, 33].

### Future research directions

Prospective multicenter observational studies are warranted to better understand the impact of diaphragmatic dysfunction on weaning success. These studies should investigate different measures of diaphragm function obtained using other modes of ultrasound [34, 35], and may consider more complex classifications of weaning success that are applicable across a diverse range of global practices [18]. Furthermore, instead of focussing just on predicting weaning success [21], these studies should use design and analysis methods to establish causality and estimate attributable patient benefits of modifiable risk factors.

The possible causal impact of providing an early dose of amino acids on preventing diaphragmatic dysfunction and reducing mortality is plausible [25, 30]. The estimates of benefit reported in this study may serve to help power a subsequent randomised controlled trial evaluating this specific effect pathway. Formal add-on studies may also be conducted on top of ongoing clinical trials currently evaluating the impact of early amino acid intake in appropriate critically ill patient populations [36].

## Conclusions

In this multicenter observational study, we found that critically ill patients who experienced diaphragmatic dysfunction may be eight times more likely to develop extubation failure and were twice as likely to die. Causal path analysis revealed that amino acid intake during the first 24 h of ICU stay could reduce the relative risk of developing diaphragmatic dysfunction by 10 to 48% and could reduce the relative risk of mortality by 25 to 73%. We recommend interventional studies to determine whether early amino acid intake can improve patient outcomes from mechanical ventilation and to identify the optimal dose of early amino acids that could result in maximum patient benefit.

## Data Availability

The datasets used and/or analysed during the current study are available from the corresponding author on reasonable request, pending ethics approval for additional analysis.

## Abbreviations

iMV: Invasive mechanical ventilation
ICU: Intensive care unit
SBT: Spontaneous breathing test
FiO_2_: Fraction of inspired oxygen
PEEP: Positive end expiratory pressure
TF: Diaphragmatic thickening fraction
APACHE: Acute physiology and chronic health evaluation
SOFA: Sequential organ failure assessment
