# Correction to: Modifiable risk factors for ventilator associated diaphragmatic dysfunction: a multicenter observational study

**DOI:** 10.1186/s12890-023-02695-y

**Published:** 2023-10-19

**Authors:** Hong Pu, Gordon S. Doig, Yu Lv, Xiaoxiao Wu, Fu Yang, Shurong Zhang, Zongan Liang, Yan Zhou, Yan Kang

**Affiliations:** 1 Department of Critical Care Medicine, West China Hospital, West China Medical School, Sichuan University, Chengdu, PR China; 2 Northern Clinical School Intensive Care Research Unit, Sydney Medical School, University of Sydney, Sydney, NSW Australia; 3 Healthcare-Associated Infection Control Center, Sichuan Academy of Medical Sciences, School of Medicine, Sichuan People’s Hospital, University of Electronic Science and Technology of China, Chengdu, PR China; 4 Department of Critical Care Medicine, Sichuan Academy of Medical Sciences, School of Medicine, Sichuan People’s Hospital, University of Electronic Science and Technology of China, Chengdu, PR China; 5 Department of Respiratory and Critical Care Medicine, West China Medical School, West China Hospital, Sichuan University, Chengdu, PR China

**Correction to: BMC Pulmonary Medicine (2023) 23:343**

10.1186/s12890-023-02633-y

Following publication of the original article [1], the authors flagged that the following declaration had been omitted from the Funding declaration of the article: Publication of the project was supported by a grant from the Key Research and Development Project of Sichuan Province Science and Technology Department (Grant No. 2021YFS0249).

The published article has since been corrected. The authors thank you for reading this article and apologize for any inconvenience caused.

